# Effect of Patient-Directed Messaging on Colorectal Cancer Screening: A Randomized Clinical Trial

**DOI:** 10.1001/jamanetworkopen.2022.4529

**Published:** 2022-03-31

**Authors:** Akinbowale Oyalowo, Kimberly A. Forde, Alicia Lamanna, Michael L. Kochman

**Affiliations:** 1 Division of Gastroenterology and Hepatology, Department of Medicine, University of Pennsylvania Perelman School of Medicine, Philadelphia; 2 Center for Clinical Epidemiology and Biostatistics, Department of Biostatistics, Epidemiology and Informatics, University of Pennsylvania Perelman School of Medicine, Philadelphia; 3 Center for Endoscopic Innovation, Research, and Training, Department of Medicine, University of Pennsylvania Perelman School of Medicine, Philadelphia

## Abstract

**Importance:**

Colorectal cancer (CRC) screening is underused in the US. Tailored message interventions have shown benefit for increasing screening uptake of mammography and Papanicolaou testing, but their role in CRC screening is less clear.

**Objective:**

To evaluate the effectiveness of a tailored message telephone intervention prior to scheduling of a screening or surveillance colonoscopy and its effect on CRC screening completion rates.

**Design, Setting, and Participants:**

This randomized clinical trial was conducted from July 2017 through August 2018 at the University of Pennsylvania Health System (UPHS), an urban academic medical center. Participants were asymptomatic patients aged 50 to 75 years who were eligible for CRC screening or surveillance, had been referred for colonoscopy, and did not have a scheduled colonoscopy appointment. Data analysis was conducted from January to September 2019.

**Interventions:**

Patients underwent block randomization in a 1:1:1 ratio to 1 of 3 study arms. Participants in the usual care group were contacted via a mailed letter and instructed to call to schedule a colonoscopy. In the generic message group, participants were contacted by telephone, completed an assessment, and received a uniform, nontailored message encouraging colonoscopy scheduling. Participants in the tailored message group were contacted by telephone, completed an assessment, and received a tailored message encouraging colonoscopy scheduling based on their identified assessment cohort.

**Main Outcomes and Measures:**

The primary outcome was colonoscopy completion rate within 120 days of enrollment. The secondary outcome was colonoscopy scheduling rate appointment within 120 days of enrollment.

**Results:**

A total of 600 participants (median [IQR] age, 56 [51-63] years; 373 women [62.2%]) were enrolled, including 200 participants randomized to usual care, 200 participants randomized to the generic message, and 200 participants randomized to the tailored message. The total sample included 12 Asian participants (2.0%), 324 Black participants (54.0%), and 227 White participants (37.8%), and 9 participants (1.5%) were of Latino or Hispanic ethnicity. Colonoscopy completion was significantly higher for both the tailored message group (69 participants [34.5%]) and the generic message group (64 participants [32.0%]) compared with the usual care group (37 participants [18.5%]) (*P* < .001 and *P* = .002, respectively). Scheduling rates were also significantly higher in both groups, with 106 participants (53.0%) in the tailored message group and 105 participants (52.5%) in the generic message group scheduling appointments, compared with 54 participants (27.0%) in the usual care arm (*P* < .001 for both).

**Conclusions and Relevance:**

In this randomized clinical trial among individuals whose CRC screening was not up to date, both a tailored message intervention and a generic message intervention were significantly more effective at increasing colonoscopy scheduling and completion rates compared with usual care. These findings suggest that individualized health communications can increase individual motivation to obtain CRC screening.

**Trial Registration:**

ClinicalTrials.gov Identifier: NCT03310892

## Key Points

**Question:**

Are individuals more likely to complete colorectal cancer screening if they receive communication that is tailored to their attitudes and beliefs?

**Findings:**

In this randomized clinical trial including 600 participants, a tailored message intervention and a generic message intervention were significantly more effective at increasing colonoscopy scheduling and colonoscopy completion rates compared with usual care. The tailored message intervention was not shown to be superior to the generic message intervention.

**Meaning:**

These findings suggest that tailored message interventions could increase CRC screening, demonstrating the importance of individualized outreach to promote cancer screening.

## Teaser

This randomized clinical trial evaluates the effectiveness of tailored or generic telephone messages in motivating patients to schedule and complete colorectal cancer screening compared with usual care.

## Introduction

Several studies have demonstrated that screening for CRC is associated with reduced incidence of CRC, as well as reduced mortality from CRC,^1,2,3,4^ and multiple national organizations have published guidelines that recommend screening individuals aged 50 to 75 years at average risk for colorectal cancer.^5,6,7^ Recently some of these groups, including the US Preventive Services Task Force, have updated their guidelines to recommend initiating screening at age 45 years in adults at average risk.^8,9,10^ Despite the significant benefits of CRC screening, rates in the US are suboptimal, with screening rates estimated to be 66.9% in 2015,^3^ well below the National Colorectal Cancer Roundtable's proposed target of 80% by 2018,^11^ and below screening rates for other cancers, such as breast cancer (72.4%) and cervical cancer (82.9%).^3^ Multiple options for CRC screening exist, including stool-based testing (ie, guaiac-based fecal occult blood test or fecal immunochemical test, alone or in combination with stool DNA examination), endoscopy (ie, flexible sigmoidoscopy or colonoscopy), and radiologic examination (ie, computed tomography colonography).^4,12^ Although no 1 strategy has been shown to have benefit over the others, colonoscopy is the most commonly used screening test in the US (61.7%).^13,14^ Furthermore, all other screening strategies require colonoscopy as follow-up to a positive test result. However, using colonoscopy as a screening strategy requires that individuals complete multiple separate steps; thus, motivation is a critical component.^15^

Several interventions to increase colorectal cancer screening rates have been studied, including the use of health-related communications to influence attitudes and intentions and thus behaviors.^16,17,18,19^ One specific evidence-based intervention is the provision of tailored health messages.^20^ Health communication interventions often lack individualization and thus may have limited influence on recipients. Some interventions have sought to personalize communication by using targeted messaging, which involves the delivery of messages to subgroups within a population based on shared characteristics, such as demographics.^21^ These communications can be a useful way to segment members of a population into groups that may respond to specific messages but do not account for varying opinions within a targeted population.^22^ In contrast, tailored message interventions use individual assessments of a person’s expressed attitudes and beliefs to deliver messages that are based on information relevant to each individual.^22,23^ A meta-analysis by Noar et al^24^ found that tailored print interventions were associated with more behavior change compared with generic information and found that tailored print behavior change interventions were associated with increasing screening uptake, including for mammography and Papanicolaou testing.

The role of tailored message interventions in CRC screening is less clear. Studies have shown benefit incorporating tailored messages compared with usual care, but to our knowledge, no study has shown a statistically significant benefit compared with other types of health communications, such as targeted messages.^23,25,26,27^ Therefore, further work is needed to better understand the role of tailored messaging in colorectal cancer screening. In this study, we evaluated the effectiveness of a tailored message telephone intervention prior to scheduling of a screening or surveillance colonoscopy and assessed its effect on CRC screening rates.

## Methods

This randomized clinical trial was approved by the University of Pennsylvania institutional review board prior to study initiation and data collection. For this trial, the institutional review board granted a waiver of informed consent because the study posed no more than minimal risk to participants, as CRC screening is the standard of care; the trial would not have been feasible if informed consent were required; and limiting the study to those who consented may have introduced selection or volunteer bias. The trial protocol and statistical analysis plan are available in Supplement 1. This study followed the Consolidated Standards of Reporting Trials (CONSORT) reporting .

### Study Design

**Figure 1.**
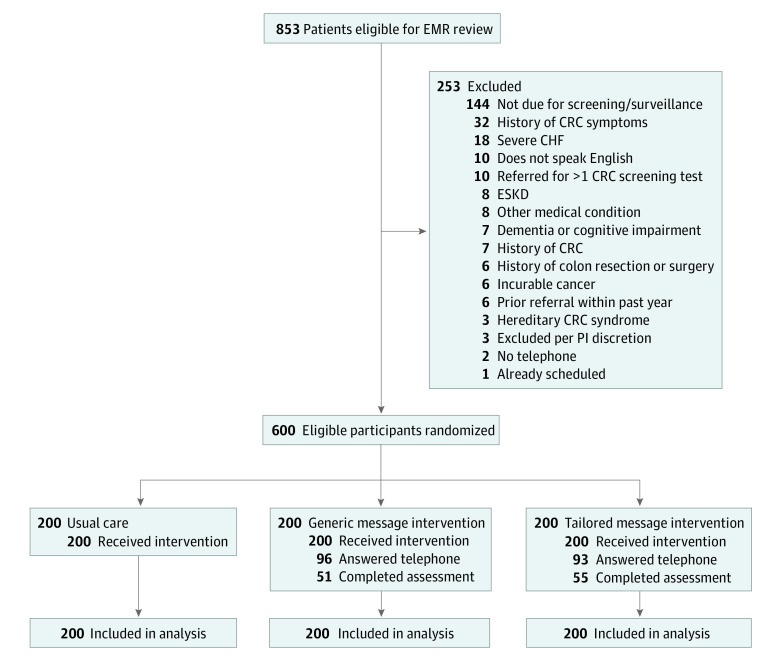
Study Recruitment Flowchart CHF indicates congestive heart failure; CRC, colorectal cancer; EMR, electronic medical record; ESKD, end-stage kidney disease; and PI, principle investigator.

This was a 3-arm randomized clinical trial. Eligible participants underwent block randomization in a 1:1:1 ratio using a computerized random number generator to 1 of 3 study groups: usual care (control), generic message telephone intervention, or tailored message telephone intervention (Figure 1). The study investigators were blinded to participant data and randomization but research staff conducting the telephone interventions were not blinded, as the interventions required staff members to deliver messages that corresponded to a participant’s assigned study group.

### Setting

The study was conducted from July 2017 through August 2018 at the University of Pennsylvania Health System (UPHS). Using the electronic medical record (EMR; Epic/PennChart), members of the research team identified UPHS primary care patients with an active referral order for screening or surveillance colonoscopy without an existing colonoscopy appointment. We included patients within the UPHS system who were aged 50 to 75 years, eligible for CRC screening or surveillance, and appropriately referred for colonoscopy by their primary care practitioner but who had not yet scheduled an appointment. We defined eligibility for CRC screening or surveillance as no record of colonoscopy in the past 5 years, fecal immunochemical testing or fecal occult blood testing in the past 12 months, flexible sigmoidoscopy or CT colonography in the past 5 years, or stool DNA test in the last 3 years. Exclusion criteria included (1) history of colorectal cancer, (2) history of inflammatory bowel disease (eg, Crohn disease or ulcerative colitis), (3) history of colon surgery or resection, (4) history of symptoms concerning for CRC (eg, lower gastrointestinal bleeding) within the past 6 months, (5) family history of a hereditary CRC syndrome (eg, familial adenomatous polyposis or hereditary nonpolyposis colon cancer), (6) current serious medical condition with estimated life expectancy of less than 6 months (eg, incurable cancer, end-stage congestive heart failure, decompensated cirrhosis, end-stage kidney disease), (7) inability to speak English, and (8) no telephone number listed in the EMR. Race and ethnicity were self-identified. We included race and ethnicity in analysis because previous literature has suggested that racial and ethnic disparities impact access and utilization of healthcare resources..

### Interventions

In prior work, our group collaborated with Mind Genomics Advisors, a consumer insight analytics firm, to develop a 7-item survey that clusters respondents into 1 of 4 distinct messaging cohorts and projects which messages are likely to be motivating or demotivating for members of a given cohort. This was performed using an experimental design that segmented individuals into distinct messaging cohorts, based on their responses to a common set of stimuli. The methods used to derive this assessment are described in eAppendix 1 in Supplement 2. This assessment was used for the generic message and tailored message telephone interventions.

Participants in the usual care group were contacted by UPHS staff via a mailed letter and given instructions to call to schedule a colonoscopy. In both intervention groups, a study team member contacted participants by telephone and administered an assessment during the conversation (eAppendix 2 in Supplement 2). After completion of the segmenting assessment, participants in the generic message group received a nontailored message encouraging colonoscopy scheduling. For participants in the tailored message group, after completion of the assessment, they received at least 1 tailored message encouraging colonoscopy scheduling based on their assessment cohort. Additional motivating statements could be offered at the staff’s discretion. In both intervention groups, participants were given the opportunity to schedule their colonoscopy appointment during the same telephone call or could call back and schedule at their own convenience. Most telephone calls were less than 5 minutes in duration. If the participant did not answer the telephone, the study team member left a voicemail message with instructions to return the telephone call. Up to 3 telephone calls were made. Intervention participants were blind to assignment of message type. Usual care participants were blind to presence of group assignment altogether. All participants were included in the data analysis.

### Outcomes

Our primary outcome was the proportion of participants who successfully completed colonoscopy within 120 days of enrollment. The secondary outcome was the proportion of patients who scheduled a colonoscopy appointment within 120 days of enrollment.

### Statistical Analysis

Based on a review of historic data from a similar patient population at UPHS, we estimated a baseline colonoscopy completion rate of 20% in the usual care group. We estimated there would be an increase in colonoscopy completion rate of at least 10 percentage points for each of the intervention groups. We recruited 600 participants (200 in each group) to have 80% power to detect an absolute 10–percentage point increase in colonoscopy screening using a conservative Bonferroni adjustment of the type 1 error rate with a 2-sided α = .025 (α = .05 of 2 possible pair-wise group comparisons). We also performed subgroup analyses on those who answered a telephone call and spoke with a study team member (189 participants), as well as those who were contacted by telephone, completed the segmenting assessment, and received a message (106 participants). Analyses were performed using χ^2^ test of proportions to compare groups using an intention-to-treat protocol. Continuous variables were compared with analysis of variance for 3-group analyses, and 2-sample *t* tests for 2-group analyses. All analyses were performed with Stata statistical software version 15.0 (StataCorp). Data analysis was conducted from January to September 2019.

## Results

### Study Population

A total of 853 potentially eligible participants were identified through the EMR (Figure 1). Using a computer-generated randomization algorithm, 600 patients (median [IQR] age, 56 [51-63] years; 373 women [62.2%]) who met inclusion criteria for participation in the study were randomly allocated in a 1:1:1 ratio to 1 of 3 study groups, including 200 participants in the usual care group, 200 participants in the generic message group, and 200 participants in the tailored message group. There were no significant differences in baseline demographic characteristics across all 3 groups (Table 1). The total sample included 12 Asian participants (2.0%), 324 Black participants (54.0%), and 227 White participants (37.8%), and 37 participants (6.2%) identified as other race, including those who listed more than 1 race or who declined to state their race; 9 participants (1.5%) were of Latino or Hispanic ethnicity. Median (IQR) household income was $44 809 ($30 797-$70 746).

**Table 1.** Baseline Demographic Characteristics by Group Assignment

Characteristic	Participants, No. (%)			
	Total (N = 600)	Usual care (n = 200)	Generic message (n = 200)	Tailored message (n = 200)
Age, median (IQR), y	56 (51-63)	56.5 (51-64)	57.5 (51.5-63.5)	56 (51-62)
Sex				
Women	373 (62.2)	127 (63.5)	118 (59.0)	128 (64.0)
Men	227 (37.8)	73 (36.5)	82 (41.0)	72 (36.0)
Race				
Asian	12 (2.0)	3 (1.5)	4 (2.0)	5 (2.5)
Black	324 (54.0)	101 (50.5)	124 (62.0)	99 (49.5)
White	227 (37.8)	85 (42.5)	60 (30.0)	82 (41.0)
Other^a^	37 (6.2)	11 (5.5)	12 (6.0)	14 (7.0)
Ethnicity				
Hispanic or Latino	9 (1.5)	2 (1.0)	2 (1.0)	5 (2.5)
Not Hispanic or Latino	591 (98.5)	198 (99.0)	198 (99.0)	195 (97.5)
Household income, median (IQR), $^b^	44 809 (30 797-70 746)	51 780 (30 797-73 227)	40 921 (29 972-70 057)	44 809 (30 797-70 746)

^a^ Other race includes those who listed more than 1 race and those who declined to state.

^b^ According to 2016 American Community Survey 5-Year Estimates for 2012 to 2016.

### Response to Intervention

**Figure 2.**
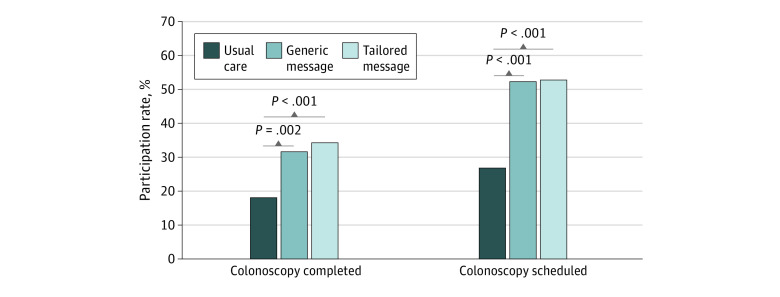
Colonoscopy Completion and Scheduling Rates by Study Group

At 120 days, 69 participants (34.5%) in the tailored message group, 64 participants (32.0%) in the generic message group, and 37 participants (18.5% ) in the usual care group had completed a colonoscopy (Figure 2). Differences were statistically significant for the tailored message vs usual care (*P* < .001) and the generic message vs usual care (*P* = .002). There was no statistically significant difference between the tailored message group and the generic message group (*P* = .60). Colonoscopy scheduling rates were also significantly higher in both intervention groups (Table 2), with 106 participants (53.0%) in the tailored message group and 105 participants (52.5%) in the generic group scheduling a colonoscopy within 120 days, compared with 54 participants (27.0%) in the usual care group (*P* < .001 for both comparisons). There was no difference in scheduling rates between the 2 intervention groups.

**Table 2.** Colonoscopy Scheduling and Completion by Subgroups

Patients	No./total No. (%)			*P* value	
	Usual care	Generic message	Tailored message	Generic vs usual care	Tailored vs usual care
**All randomized**					
Scheduled colonoscopy	54/200 (27.0)	105/200 (52.5)	106/200 (53.0)	<.001	<.001
Completed colonoscopy	37/200 (18.5)	64/200 (32.0)	69/200 (34.5)	.002	<.001
**Answered telephone**					
Scheduled colonoscopy	NA	62/96 (64.6)	53/93 (57.0)	NA	NA
Completed colonoscopy	NA	34/96 (35.4)	28/93 (30.1)	NA	NA
**Completed assessment**					
Scheduled colonoscopy	NA	39/51 (76.5)	41/55 (74.5)	NA	NA
Completed colonoscopy	NA	19/51 (37.3)	22/55 (40.0)	NA	NA

Abbreviation: NA, not applicable.

### Subgroup Analysis

Of 200 participants in the generic message group, 96 (48.0%) answered a telephone call and spoke to a study team member. In the tailored message group, 93 of 200 participants (46.5%) answered a telephone call. More than one-fourth of all intervention participants completed the full assessment and received a tailored or generic message, with 51 of 200 participants (25.5%) in the generic message group and 55 of 200 participants (27.5%) in the tailored message group completing the assessment.

For both subgroups, we determined the proportion of patients who successfully completed colonoscopy and successfully scheduled colonoscopy within 120 days of enrollment (Table 2). In the subgroup of participants who answered a telephone call, 34 participants (35.4%) in the generic message group and 28 participants (30.1%) in the tailored group arm completed a colonoscopy, with no statistically significant difference in the colonoscopy completion rate for the tailored message group compared with usual care, but all other differences between the intervention groups and usual care were statistically significant.

Among patients who completed the assessment, 19 participants (37.3%) in the generic message group and 22 participants (40.0%) in the tailored message group completed a colonoscopy. Furthermore, 39 participants (76.5%) in the generic message group and 41 participants (74.5%) in the tailored intervention group scheduled colonoscopy In this subanalysis, differences between the intervention groups and usual care were statistically significant, but the intervention groups did not differ from each other (Table 2).

Less than half of all participants (265 participants [44.2%]) scheduled a colonoscopy within the study period. Among participants who scheduled a colonoscopy, 170 (64.2%) completed the colonoscopy within the study period. In this subanalysis, the differences in completed colonoscopy among those with scheduled colonoscopy between the intervention groups and usual care were not statistically significant (Table 3).

**Table 3.** Colonoscopy Completion Among Participants Who Scheduled a Colonoscopy

Outcome	No./No. (%)				*P* value	
	Total	Usual care	Generic message	Tailored message	Generic vs usual care	Tailored vs usual care
Scheduled colonoscopy	265/600 (44.2)	54/200 (27.0)	105/200 (52.5)	106/200 (53.0)	<.001	<.001
Completed colonoscopy	170/265 (64.2)	37/54 (68.5)	64/105 (61.0)	69/106 (65.1)	NA	NA
Failed to complete colonoscopy	95/265 (35.8)	17/54 (31.5)	41/105 (39.0)	37/106 (34.9)		

Abbreviation: NA, not applicable.

## Discussion

In this randomized clinical trial, we found that among UPHS patients due for CRC screening or surveillance, a tailored message intervention and a generic message intervention were both significantly more effective at increasing colonoscopy scheduling and colonoscopy completion rates at 120 days compared with usual care. The findings of this study are similar to results from prior studies and contribute to the small yet developing literature examining tailored message interventions to increase CRC screening. A 2007 study^26^ found that both targeted and tailored interventions were associated with increased colorectal cancer screening use in a primary care practice setting. More recently, a 2016 randomized clinical trial^28^ showed that a tailored message intervention was significantly more effective compared with usual care but was not more effective than a generic message.

In this study, there were no statistically significant differences between the tailored message and the generic message interventions. There are possible explanations why this study did not show a difference between the tailored message intervention and the generic message intervention. Speaking on the phone with dedicated research staff and completing the assessment may have increased participants’ motivation to participate in CRC screening, independent of delivery of a tailored message. As demonstrated in the subgroup analysis, more participants in both intervention groups scheduled colonoscopy after engaging in a telephone call or completing the assessment. Thus, the study may have been underpowered to detect the effect from the tailored message itself. Additionally, interactions with participants took place with a study team member rather than a health care professional with a preexisting relationship with the patient. Given that the assessment and message were delivered via telephone, it is possible that some patients would be more receptive to complete CRC screening if they had a relationship with the person administering the assessment or participated in a longer discussion. Previous research by Lucas et al^29^ has reported that loss-framed messaging may reduce receptivity to CRC screening among Black patients by increasing perceived racism, and a 2014 study by Resnicow et al^30^ reported that Black individuals may prefer a more directive communication style from their health care practitioners than White individuals.

Our study showed that there was an increase in the proportion of participants who scheduled colonoscopy based on the amount of interaction with the study team: 53% of all participants in the intervention groups scheduled a colonoscopy within 120 days, and this proportion increased to 61% among those who answered a telephone call and 75% for those who completed the segmenting assessment. Despite the increase in scheduling rates when analyzed by subgroups, colonoscopy completion rates remained at 40% or lower, even for the subgroup that completed the segmenting assessment. Among all participants, 265 patients scheduled a colonoscopy, but only 170 participants (64%) completed their colonoscopy within 4 months. These findings suggest that individualized health communications can increase patient motivation to obtain a CRC screening test, as has been shown in other preventive health settings.^20,31,32^ In contrast, outreach interventions that lack tailored messaging may not be sufficient to change intentions and behavior.^33^ The results of this study also suggest that additional barriers can limit CRC screening completion despite individual intentions, given that 36% of participants did not complete their scheduled colonoscopy within the study period. Multiple factors may have contributed to lack of colonoscopy completion in our study population, including access to a timely appointment, out-of-pocket costs, transportation limitations, inadequate bowel preparation, and changes in health status. The discrepancy between colonoscopy scheduling rates and colonoscopy completion rates calls attention to the need for complementary interventions, such as patient navigation assistance, to ensure that scheduled patients actually obtain their colonoscopy in a timely manner.^34,35^ The effect of health communication interventions on CRC screening rates will be limited if patients cannot easily access their screening test of choice after referral.

### Limitations and Strengths

There were limitations in this study. This study was conducted within a single health system, so the results may not be generalizable to a patient population with different demographics than those at UPHS. Both the generic message and tailored message interventions were limited to those participants who answered a telephone call from a study member and completed the assessment; therefore, not all participants completed all steps of the intervention. Individuals who were not reached by telephone or who chose to decline the telephone assessment may have differed from those who did complete a telephone intervention. Additionally, the telephone call may not be the best medium to administer the assessment and deliver health-related messages. Future interventions may benefit from other modes of message delivery, such as web-based materials or text messaging. For this study, we used a modified assessment of 7 items on a 3-point scale to facilitate easy administration. By doing so, we may have lost some accuracy in identifying motivating or demotivating messages and might have assigned some participants to an inaccurate messaging cohort. We attempted to account for this by excluding any statements that were identified to be demotivating across all messaging cohorts.

This study also has strengths, including its prospective design and randomization at the patient level. As a randomized clinical trial, it demonstrates how outreach could be implemented in combination with usual clinical care, which could make it generalizable to many practice settings. The study design also helped avoid volunteer bias, since participants were enrolled after their heath care practitioner already made referrals for colonoscopy and the requirement of informed consent for participants was waived. This study enrolled a racially and socioeconomically diverse patient population, and most participants in our study population were Black, which is notable since Black patients are more likely to have lower rates of CRC screening and worse colon cancer outcomes.^36^ This study offers an approach to screening outreach that may help reduce racial and ethnic disparities in CRC screening.

## Conclusions

This randomized clinical study found that tailored and generic message interventions for segmented individuals increased colonoscopy rates compared with usual care. Although the tailored message intervention was not shown to be superior to a generic message intervention, it remains important to individualize health-related messages and deliver patient-centered care to increase positive health behaviors, such as CRC screening.

## Notes

Supplement 1.
Trial Protocol and Statistical Analysis Plan

Supplement 2. **eAppendix 1.** Methods to Develop Segmenting Assessment **eAppendix 2.** Script for Telephone Intervention

Supplement 3.
Data Sharing Statement
