# Supplementary material for: Effect of Patient-Directed Messaging on Colorectal Cancer Screening: A Randomized Clinical Trial
Source: JAMA Netw Open. 2022 Mar 31;5(3):e224529. doi: 10.1001/jamanetworkopen.2022.4529 (PMC8972032; doi:10.1001/jamanetworkopen.2022.4529)
Supplement: Supplement 1. — Trial Protocol and Statistical Analysis Plan [file jamanetwopen-e224529-s001.pdf]

Social and Behavioral Sciences Human Research Protocol Template

October 3, 2017

PRINCIPAL INVESTIGATOR: Michael Kochman, MD

PROTOCOL TITLE: Patient-directed messaging to increase colorectal cancer screening

INTRODUCTION AND PURPOSE:

The proposed study is a prospective, randomized trial. All eligible subjects (patients identified to be called to schedule screening colonoscopy) will be randomized to one of three study arms: usual scheduling process (control), a generic message arm, or a tailored message arm. Patients in the "tailored message" arm will receive a telephone call and be asked a series of questions that will be used to assign patients to one of four messaging cohorts. After these questions have been answered, the patient will then receive a tailored message corresponding to his or her respective messaging cohort, encouraging them to schedule a colonoscopy with a directed script. In the "generic message" arm, patients will receive a telephone call and be asked to answer the same series of questions as the “tailored message” group, then receive a single, standard script encouraging them to schedule a colonoscopy.

OBJECTIVES:

Primary Objective: To determine whether direct messaging provided to patients prior to scheduling colonoscopy will increase patient adherence to physician recommendation for screening or surveillance colonoscopy.

Secondary Objective: To determine whether direct messaging provided to patients prior to scheduling colonoscopy will increase the rate at which patients schedule a screening or surveillance colonoscopy after a recommendation from their physician.

BACKGROUND:

Colorectal cancer (CRC) is the second leading cause of cancer-related deaths in the United States among men and women [1]. In 2017, there will be an estimated 135,430 new cases of CRC diagnosed in the US, with approximately 50,260 deaths from the disease. It has been shown that screening for CRC reduces the incidence of CRC, as well as mortality from CRC [2]. Despite the significant benefits, the rate of CRC screening is suboptimal. In 2012, only 65% of all eligible adults were up-to-date with CRC screening [3] and nearly 28% had never obtained any screening test for CRC, well below the The National Colorectal Cancer Roundtable's proposed target of 80% CRC screening rate by the year 2018 [4].

Several interventions to increase CRC screening rates have been studied, including the role of health-related communications to persuade certain groups within a population [5]. Some interventions have relied on targeted messaging, which involves the delivery of messages to subgroups within a population, often based on demographics. Common characteristics of a group within a population can be identified and a message consistent with those characteristics is delivered [6]. This approach assumes that if group members possess enough similar characteristics and motivations, they will be influenced by the same message content. These interventions, however, do not account for varying opinions within a targeted population, thus the message may not have the same significance to each individual member.

Tailored interventions are intended to reach one specific person, based on characteristics that are unique to that person [7]. Tailoring relies on a personal assessment and uses that measured personal information to deliver messages that are directly relevant to a person's expressed attitudes and beliefs [7,8]. Since messages are based on personal information, it is hypothesized to be more likely to influence the desired behavior.

CHARACTERISTICS OF THE STUDY POPULATION:

1. Target Population and Accrual:

Patients will be recruited from the University of Pennsylvania Health System (UPHS). In this study, members of the research team will identify patients who are at average risk for developing colorectal cancer and have been referred for a screening or surveillance colonoscopy but have not yet scheduled the procedure. We will randomize some of these eligble patients to usual care where patients are contacted by the UPHS call center and given the opportunity to schedule their procedure during the telephone call. Other eligible patients will be randomized to a group of patients who will receive a phone call from a member of the study team and receive a message encouraging colonoscopy scheduling and will be offered the opportunity to schedule the procedure during the same call.

2. Key Inclusion Criteria:

1. UPHS patients with an active order for screening or surveillance colonoscopy without an existing colonoscopy appointment
2. 50-75 years of age
3. Due for CRC screening or surveillance (defined as no evidence of: colonoscopy in the past 5 years, fecal immunochemical testing in the past 12 months, flexible sigmoidoscopy or CT colonography in the past 5 years, stool DNA test or Cologuard in the last 3 years)

3. Key Exclusion Criteria:

1. Age <50 or >75
2. Pregnant woman
3. Evidence of prior colonoscopy in the past 5 years, fecal immunochemical testing in the past 12 months, flexible sigmoidoscopy or CT colonography in the past 5 years, fecal DNA test or Cologuard in the last 3 years
4. History of colorectal cancer
5. History of inflammatory bowel disease (Crohn’s disease or Ulcerative colitis)
6. History of colon surgery or resection
7. History of symptoms concerning for colorectal cancer, such as lower GI bleeding, within the past 6 months
8. Family history of a hereditary colorectal cancer syndrome, such as familial adenomatous polyposis (FAP) or hereditary non-polyposis colon cancer (HNPCC)
9. Current serious medical condition with estimated life expectancy of less than 6 months, such as incurable cancer, end-stage congestive heart failure, decompensated cirrhosis, end stage renal disease, etc.
10. Dementia
11. Does not speak English
12. No telephone number listed in electronic medical record
13. Has any other condition that, in the opinion of the investigator, excludes the patient from participating in this study

4. Subject Recruitment and Screening:

Subjects will be recruited using the electronic medical record to identify all patients at UPHS with an order for colonoscopy with a listed indication of screening or surveillance. Once a potential participant is identified, that individual will be contacted by telephone in accordance with the current operating procedures at the UPHS call center. Advertisements with Penn Media services will not be required for patient outreach.

5. Early Withdrawal of Subjects:

Subjects may withdraw from the study at any time without any impact to their care. This can be done by choosing not to respond to telephone call from study member, terminating telephone call early, or requesting to be excluded from study participation.

Subjects may also be discontinued from the study at the discretion of the Investigator if there is a change in eligibility. It will be documented whether or not each subject completes the study. For those who elect to withdraw from the study, they will be asked for permission to have the study team review their chart to determine if and when colonoscopy has been completed. If this permission is not granted, their information will be removed from the study.

6. Vulnerable Populations:

N/A

7. Populations vulnerable to undue influence or coercion:

N/A

STUDY DESIGN:

METHODS:

1. Study Instruments:

The primary endpoint is the rate of colonoscopy completion within 120 days from randomization.

The secondary endpoint is the rate of scheduling completion in each of the three groups, defined as the ratio of the number of participants who elect to schedule a colonoscopy to the total number of participants contacted by telephone or in receipt of letter from UPHS and asked to schedule a colonoscopy.

The electronic medical record will be accessed to identify all patients at UPHS with an active order for colonoscopy with a listed indication of screening or surveillance. For each participant, the study starts at the time of randomization. The study end date will be date of colonoscopy completion or 120 days from randomization, whichever comes first.

2. Group Modifications:

All patients will have the opportunity to schedule a colonoscopy at their convenience. For patients in arm 2 and arm 3 of the study, we will ask patients to answer a series of questions used to cluster patients into one of four messaging cohorts, based on their responses. After answering questions, all patients in arm 2 will receive the same "generic" message encouraging the patient to schedule CRC screening, regardless of their responses to the preceding questions. Patients in arm 3 of the study will receive a "tailored" message encouraging colonoscopy scheduling based on their assigned messaging cohort, which will be determined by their responses to the preceding questions.

3. Method for Assigning Subjects to Groups:

All subjects will be randomized in 1:1:1 ratio using a computer-generated randomization algorithm to one of three arms: a control arm (usual scheduling process), a generic message arm, or a tailored message/intervention arm.

4. Administration of Surveys and/or Process:

Subjects randomized to one of the two telephone message arms will be contacted by telephone, asked to answer a series of questions, then receive a generic or tailored message encouraging CRC screening. We anticipate the telephone call will not last more than 10-15 minutes. The research staff will make up to three attempts to speak directly with the subject. There are no further communications between the subject and research team after the telephone call.

5. Data Management:

Information about study subjects will be kept confidential and managed according to the requirements of the Health Insurance Portability and Accountability Act of 1996 (HIPAA). Source documents are maintained in the UPHS EMR (Epic/PennChart). No source documents will be printed or maintained in paper form at the study site. Data from Epic/PennChart will be recorded in a secure database system. The study team will have access to PHI within Epic/PennChart and database. All PHI within the database will be labelled as identifiable information so that only de-identified exports are possible. All reports that include identifiable information will be stored on a secure drive, maintained behind the UPHS firewall. Once data analysis and manuscripts have been published, the data from the database will be removed and de-identified on the secure drive. This de-identified dataset will be stored for up to five years after analysis is complete and manuscripts have been published. Once analysis is completed and any manuscripts are published, we will retain PHI no longer than seven years in accordance with government regulations, applicable policies, and institutional requirements.

6. Subject Follow-up:

Visits are not required for this study. If a subject requests to schedule a colonoscopy at the conclusion of the phone call, and is randomized to one of the intervention arms but does not answer telephone call initially, up to two additional attempts will be made.

STUDY PROCEDURES:

1. Detailed Description:

Screening:

We will submit a data request for all patients within the UPHS electronic medical record (Epic/PennChart) that have been referred for a screening or surveillance colonoscopy, but have not yet scheduled their procedure. For each potential study subject identified via this query, the research team will review that individual’s chart in Epic/PennChart to confirm study eligibility. We will continue this process until at least 495 subjects have been enrolled and randomized.

Randomization:

Using a computer-generated randomization algorithm, all eligible subjects will undergo randomization in a 1:1:1 ratio to one of three groups: 1. usual care 2. telephone call by study member with delivery of generic message 3. telephone call by study member with delivery of tailored message. Subjects in the usual care group will not receive any contact from the research team. The research coordinator will record the randomization assignments on a master list which will be maintained on a password protected computer in a locked office.

Study Intervention:

The study team will contact subjects randomized to arms 2 and 3. If a subject does not answer a telephone call during the first attempt, up to two additional attempts will be made. Once a subject answers the call, study team will begin conversation with identical opening language used by the UPHS call center. The subject will then be asked to answer a series of 7 questions (see attached script). After the responses have been recorded, the research member will deliver a corresponding message to the subject depending on the arm to which the subject has been randomized to. Subjects will then be asked to schedule a colonoscopy during the telephone call. If they respond “yes”, they will be transferred to a separate line for scheduling.

Follow-Up:
Once a patient is randomized to one of the three arms, he/she will be enrolled in the study, even if the telephone call is not completed or the patient declines colonoscopy scheduling at that time. A subject's participation in the study ends once the subject completes a colonoscopy or 120 days from initial telephone contact or randomization, whichever comes first.

2. Data Collection:
See study description above

3. Genetic Testing:
N/A

4. Use of Deception:
N/A

5. Statistical Analysis:
We estimate a baseline colonoscopy completion rate of 20% in arm 1 (Call Center alone). We estimate an increase in colonoscopy completion rate of at least 10 percentage points for the intervention arms. By recruiting at least 600 participants (200 in each arm), we will have 80% power to detect an absolute 10 percentage point increase using a conservative Bonferroni adjustment of the Type 1 error rate with a two-sided alpha level of 0.025. Analysis will be conducted by blinded members of the research team.

RISK/BENEFIT ASSESSMENT:

1. Risks:
The risks associated with this study are no more than minimal risk. There is the potential risk of breach of confidentiality. We will minimize this risk by using de-identified information whenever possible and by maintaining all identifiable information on a secure drive and/or in a HIPAA-compliant system. There is a possible risk of psychological harm associated with encouragement to schedule a colonoscopy to screen for CRC. This risk is minimized by facilitating timely scheduling of the colonoscopy, and UPHS providers who perform the colonoscopy will communicate the results of the procedure and coordinate subsequent management accordingly.

2. Benefits:
Individual subjects may benefit from participation in this study if randomization to an intervention arm increases the likelihood of scheduling and completing a screening or surveillance colonoscopy. Data generated from this study may benefit society and the research community by adding further knowledge of how to effectively increase CRC screening rates.

3. Subject Privacy:
Our only interaction with subjects will be the subset who are randomized to receive a telephone call from the study team. With these subjects, we will conduct phone calls in a private area and verify the subject's identify before delivering any message. We will not interact with subjects in person.

4. Subject Confidentiality:

How will confidentiality of data be maintained? Check all that apply.

☒ Paper-based records will be kept in a secure location and only be accessible to personnel involved in the study.

☒ Computer-based files will only be made available to personnel involved in the study through the use of access privileges and passwords.

☒ Prior to access to any study-related information, personnel will be required to sign statements agreeing to protect the security and confidentiality of identifiable information.

☒ Whenever feasible, identifiers will be removed from study-related information.

☐ A Certificate of Confidentiality will be obtained, because the research could place the subject at risk of criminal or civil liability or cause damage to the subject's financial standing, employability, or liability.

☐ A waiver of documentation of consent is being requested, because the only link between the subject and the study would be the consent document and the primary risk is a breach of confidentiality. (This is not an option for FDA-regulated research.)

☒ Precautions are in place to ensure the data is secure by using passwords and encryption, because the research involves web-based surveys.

☐ Audio and/or video recordings will be transcribed and then destroyed to eliminate audible identification of subjects.

☐ Other (specify):

Information about study subjects will be kept confidential and managed according to the requirements of the Health Insurance Portability and Accountability Act of 1996 (HIPAA). Please refer to our request to waive HIPAA authorization. We will be using de-identified information whenever possible and maintain all identifiable information on a secure drive and/or in a HIPAA-compliant system.

5. Protected Health Information

- ***Name***
- ***Street address, city, county, precinct, zip code, and equivalent geocodes***
- ***All elements of dates (except year) for dates directly related to an individual and all ages over 89***
- ***Telephone numbers***
- ***Fax numbers***
- ***Electronic mail addresses***
- *Social security numbers*
- ***Medical record numbers***
- ***Health plan ID numbers***
- *Account numbers*
- *Certificate/license numbers*
- *Vehicle identifiers and serial numbers, including license plate numbers*
- *Device identifiers/serial numbers*
- *Web addresses (URLs)*
- *Internet IP addresses*
- *Biometric identifiers, including finger and voice prints*
- *Full face photographic images and any comparable images*
- *Any other unique identifying number, characteristic, or code*

6. Compensation:

There is no compensation for participation in this study.

7. Data and Safety Monitoring:

Safety will be monitored on an ongoing basis by the PI and the study team. The PI or designee will review the study charts to evaluate events at each subject interaction to ensure the grade, relationship to the study procedure, expectedness and the course of action for each subject is documented. The PI or Sub- investigator is ultimately responsible for assigning grade and attribution.

8. Investigator's Risk/Benefit Assessment:

The risks associated with this study are no more than minimal risk. Data from this study will help identify how tailored messages may motivate or discourage groups of individuals to obtain a screening test for CRC, with the aim of increasing CRC screening rates. For these reasons and those outlined in the above sections, the Principal Investigator believes that the risks of participating in the study are outweighed by the potential benefits of participating in the study.

INFORMED CONSENT:

1. Consent Process:

We request a waiver of informed consent (see below).

2. Waiver of Informed Consent:

<u>Minimal Risk</u>

This study involves no more than minimal risk to subjects. The only interventions in this study are additional messaging to attempt to increase colonoscopy adherence. All subjects are patients who have received a referral for screening colonoscopy by their physician or advance practice provider. Participation in this study would involve randomization of subjects to different methods for contacting patients to schedule colonoscopy to assess whether a telephone message can influence likelihood that a patient schedules and completes a colonoscopy. For many health systems, including UPHS, telephone messages to patients at time of scheduling a screening colonoscopy is standard of care. The addition of consent may reduce our response rate and potentially decrease the rate of colonoscopy scheduling and/or completion.

<u>Impact on Subject Rights and Welfare</u>

Subjects' rights and welfare will not be adversely affected by the waiver of authorization and consent. Although subjects will be randomized to different outreach methods, this does not impair subjects' rights and welfares because all subjects will be

able to choose to schedule a screening or surveillance colonoscopy at their convenience or decline scheduling. Certain subjects will be randomized to receive additional encouragement for scheduling via telephone call, but subjects can decline this call or terminate the call at any point. Identifying which outreach method is most effective has societal benefits (by increasing the rate of colorectal cancer screening) and requires waiver of informed consent to avoid selection bias.

Waiver Essential to Research

If informed consent is required, we believe that it would potentially create a selection bias, as those patients who consent to participate may also be patients who are more likely to schedule colonoscopy. Waiving documentation of consent alone would not eliminate this issue. Furthermore, we believe that the inclusion of informed consent may discourage some patients from scheduling a colonoscopy at all, thus reducing the number of patients who schedule a clinically indicated procedure.

RESOURCES NECESSARY FOR HUMAN RESEARCH PROTECTION:

The principal investigator, Michael Kochman, is the Wilmott Family Professor of Medicine at the Perelman School of Medicine, University of Pennsylvania. All members of the research team have completed CITI human subjects research training.

REFERENCES

1. Siegel RL, Miller KD, Jemal A. Cancer Statistics, 2017. CA Cancer J Clin. 2017;67:7-30.

2. Lin JS, Piper MA, Perdue LA, et al. Screening for Colorectal Cancer: Updated Evidence Report and Systematic Review for the US Preventive Services Task Force. JAMA. 2016;315:2576-2594.

3. Centers for Disease Control and Prevention (CDC). Vital signs: colorectal cancer screening test use--United States, 2012. MMWR Morb Mortal Wkly Rep. 2013;62:881-888.

4. National Colorectal Cancer Roundtable. http://nccrt.org/tools/80-percent-by-2018/ Accessed on 7/6/2017.

5. Albada A, Ausems MG, Bensing JM, van Dulmen S. Tailored information about cancer risk and screening: a systematic review. Patient Educ Couns. 2009;77:155-171.

6. Schmid KL, Rivers SE, Latimer AE, Salovey P. Targeting or Tailoring? Maximizing Resources to Create Effective Health Communications. Mark Health Serv. 2008;28:32-37.

7. Kreuter MW, Strecher VJ, Glassman B. One size does not fit all: the case for tailoring print materials. Ann Behav Med. 1999;21:276-283.

8. Vernon SW, Bartholomew LK, McQueen A, et al. A randomized controlled trial of a tailored interactive computer-delivered intervention to promote colorectal cancer screening: sometimes more is just the same. Ann Behav Med. 2011;41:284-299.
