# Supplementary material for: Effect of Patient-Directed Messaging on Colorectal Cancer Screening: A Randomized Clinical Trial
Source: JAMA Netw Open. 2022 Mar 31;5(3):e224529. doi: 10.1001/jamanetworkopen.2022.4529 (PMC8972032; doi:10.1001/jamanetworkopen.2022.4529)
Supplement: Supplement 2. — eAppendix 1. Methods to Develop Segmenting Assessment eAppendix 2. Script for Telephone Intervention [file jamanetwopen-e224529-s002.pdf]

Supplemental Online Content

Oyalowo A, Forde KA, Lamanna A, Kochman ML. Effect of patient-directed messaging on colorectal cancer screening: a randomized clinical trial. *JAMA Netw Open*. 2022;5(3):e224529. doi:10.1001/jamanetworkopen.2022.4529

eAppendix 1. Methods to Develop Segmenting Assessment
eAppendix 2. Script for Telephone Intervention

This supplemental material has been provided by the authors to give readers additional information about their work.

© 2022 Oyalowo A et al. *JAMA Network Open.*

eAppendix 1. Methods to Develop Segmenting Assessment

We collaborated with Mind Genomics Advisors (MGA), a consumer insight analytics firm, to develop an assessment tool that identifies individual preferences by population cohort group. Using a market research panel provider, individuals from a national sample were invited to complete an online survey and compensated for participation. Respondents were eligible for inclusion in the study if they met the following criteria: men and women with an age of 50-75 years, a total family household income <$40,000 annually, current medical insurance, and self-identification as Black or African American. These criteria were selected to reflect the population of patients enrolled in an existing CRC patient navigation program at the University of Pennsylvania Health System.

Using a 9-point scale, each participant rated a unique set of 48 vignettes on the following question: “How likely are you to get a screening test to prevent Colon Cancer based on this information?” Three or four messaging statements were included in each vignette. This design ensured that each element appeared an equal number of times and each element was statistically independent of every other element.

Their responses were used to cluster subjects into distinct segments. Regression analysis was performed to quantify the incremental appeal of each message relative to a baseline level of response. We related the 36 independent variables, the messaging statements, to the binary dependent variable, likelihood to obtain a screening test.

© 2022 Oyalowo A et al. *JAMA Network Open.*

A coefficient score was assigned to each message, indicating the ability of that message to influence a subject independent of the influence of any other message. Our data suggest four distinct segments representing groups of respondents likely to respond differently to specific messaging. The findings from this study led to the subsequent development of a web-based, seven-item survey that clusters respondents into one of the four messaging cohorts to facilitate easy administration of the assessment.

© 2022 Oyalowo A et al. *JAMA Network Open.*

eAppendix 2. Script for Telephone Intervention

[Caller makes introduction, confirms patient information]

In order to best assist you, I will read you seven statements. I would like you to tell me **how likely you are to get a screening test to prevent colon cancer** based on each statement. Your answer choices are **"Not At All Likely", "Unsure", or "Very Likely".**

Statement 1: Early diagnosis of colon cancer can often lead to a complete cure

Statement 2: Getting colon cancer screening is nothing to be embarrassed about

Statement 3: Colon cancer screening is absolutely need in catching and stopping colon cancer early

Statement 4: You have concerns about getting a colon cancer screening

Statement 5: The primary goal of colon cancer screening is to prevent death from colorectal cancer

Statement 6: Your body is a temple of God. You can prevent colon cancer by getting a screening

Statement 7: Live a healthy life to see your grandchildren get married

Enter the responses into the assessment tool. Subject's viewpoint should now be identified. Proceed to corresponding page for prompts.

© 2022 Oyalowo A et al. *JAMA Network Open.*

Viewpoint 1: "I Am Convinced"

[Note for reader: This viewpoint is motivated to obtain a screening test to prevent colon cancer. There are no motivating or demotivating messages. Encourage scheduling, but if the subject needs convincing, the following statements may help.]

Motivating statements

The primary goal of colon cancer screening is to prevent death from colorectal cancer

Screening for colorectal cancer may save your life

You would like to follow your doctor's advice, and this includes you having a colon cancer screening test

Early diagnosis of Colon Cancer can often lead to a complete cure

Delaying a colon cancer screening will do more harm than good

Colon Cancer screening is covered by your insurance company

Colon Cancer screening is absolutely needed in catching and stopping colon cancer early

© 2022 Oyalowo A et al. *JAMA Network Open.*

Viewpoint 2: "Screening & Prevention"

[Note for reader: This viewpoint is motivated by statements promoting the benefits of screening. Discuss how screening can prevent death from colon cancer because once symptoms of colon cancer appear, it is often too late for a cure. Avoid using statements about risk factors for colon cancer or personal perspective.]

<u>Motivating statements</u>

The primary goal of colon cancer screening is to prevent death from colorectal cancer

Screening for colorectal cancer may save your life

Colon Cancer screening is absolutely needed in catching and stopping colon cancer early

Colon Cancer screening is covered by your insurance company

To prevent Colon Cancer... get a screening test

<u>AVOID these statements</u>

Colon Cancer starts from a small growth called a polyp… these are painlessly removed during the colonoscopy and eliminates the cancer

In the United States, colorectal cancer is one of the leading causes of deaths due to cancer

Live to see your children and grandchildren raise their families

There is no shame in getting a Colon Cancer Screening Test

Live a healthy life to see your grandchild get married

You have a high risk of colon cancer if you have a family history of colon cancer

You have a high risk of colon cancer if you have a personal history of breast cancer

© 2022 Oyalowo A et al. *JAMA Network Open.*

Viewpoint 3: “Appeal & Early Detection For A Cure”

[Note for reader: This viewpoint is motivated when the messages relate to a personal perspective and early detection to increase chances of a cure. Avoid discussing the process of screening or the benefits of screening]

Motivating statements

Delaying a colon cancer screening will do more harm than good

Early diagnosis of Colon Cancer can often lead to a complete cure

Your body is a temple of God… prevent Colon Cancer by getting a screening

Colon Cancer screening is absolutely needed in catching and stopping colon cancer early

Delaying a colon cancer screening will do more harm than good

AVOID these statements

Colorectal cancer can be prevented with a screening colonoscopy

Colon cancer screening is painless… you sleep through the test

In the United States, colorectal cancer is one of the leading causes of deaths due to cancer

There is a preparation for Colon Cancer screening that is just right for you

Getting a colon cancer screening is nothing to be embarrassed about

Eliminate worrying about colon cancer… get a Colon Cancer screen test now and get peace of mind

© 2022 Oyalowo A et al. *JAMA Network Open.*

Viewpoint 4: "Convinced…Don't Tell Me About Risks"

[Note for reader: This viewpoint is highly motivated to get a screening test but is also easily de-motivated by several types of messages. Avoid discussing risk factors, death and concerns related to colon cancer screening.]

Motivating statements

You would like to follow your doctor's advice, and this includes you having a colon cancer screening test

AVOID these statements

There is no shame in getting a Colon Cancer Screening Test

When you know nothing about Colon Cancer Screening… we have an expert at the hospital to answer all your questions

Live a healthy life to see your grandchild get married

You have a high risk of colon cancer if you are African American

Colon Cancer often happens without any warning

In the United States, colorectal cancer is one of the leading causes of deaths due to cancer

Do what is best for your family… get a colon cancer screening

You have a high risk of colon cancer if you have a personal history of breast cancer

When someone in your family has had colon cancer, you are at higher risk and need to have a colon cancer screening test NOW

You have a high risk of colon cancer if you have a family history of colon cancer

Colon Cancer symptoms often appear too late to cure it

You have concerns about getting a Colon Cancer Screening…Speak to someone that you trust to eliminate your concerns

Don't let your loved ones see you die from colon cancer…get a screening test early

© 2022 Oyalowo A et al. *JAMA Network Open.*
