# Supplementary material for: Effect of Patient-Directed Messaging on Colorectal Cancer Screening: A Randomized Clinical Trial
Source: JAMA Netw Open. 2022 Mar 31;5(3):e224529. doi: 10.1001/jamanetworkopen.2022.4529 (PMC8972032; doi:10.1001/jamanetworkopen.2022.4529)
Supplement: Supplement 3. — Data Sharing Statement [file jamanetwopen-e224529-s003.pdf]

Data Sharing Statement

Oyalowo. Effect of Patient-Directed Messaging on Colorectal Cancer Screening. *JAMA Netw Open*. Published March 31, 2022. doi:10.1001/jamanetworkopen.2022.4529

Data
Data available: No

Additional Information
Explanation for why data not available: not applicable for review
